# HIV testing practices among New England college health centers

**DOI:** 10.1186/1742-6405-10-8

**Published:** 2013-03-18

**Authors:** Nilay Patel, Aadia Rana, Alyssa Thomas, John C Barnhart, Timothy P Flanigan, Jacob J van den Berg, Philip A Chan

**Affiliations:** 1 Alpert Medical School of Brown University, Division of Infectious Diseases, The Miriam Hospital, 1125 North Main Street, Providence, RI 02904, USA

**Keywords:** HIV, College, STI, Prevention

## Abstract

**Background:**

The prevalence of human immunodeficiency virus (HIV) continues to increase among certain populations including young men who have sex with men (MSM). College campuses represent a potential setting to engage young adults and institute prevention interventions including HIV testing. The purpose of this study was to evaluate testing practices for HIV and other sexually transmitted infections (STIs) on college campuses.

**Methods:**

Medical directors at four-year residential baccalaureate college health centers in New England were surveyed from June, 2011 to September, 2011. Thirty-one interviews were completed regarding experiences with HIV testing, acute HIV infection, other STI testing, and outreach efforts targeting specific at-risk groups such as MSM.

**Results:**

Among schools that responded to the survey, less than five percent of students were tested for HIV at their local college health center in the past academic year (2010–2011). Significant barriers to HIV testing included cost and availability of rapid antibody testing. One-third of college health medical directors reported that their practitioners may not feel comfortable recognizing acute HIV infection.

**Conclusions:**

Improved HIV testing practices are needed on college campuses. Programs should focus on outreach efforts targeting MSM and other at-risk populations.

## Background

The human immunodeficiency virus (HIV) epidemic has continued unabated in the United States with approximately 48,600 new cases per year, prompting a nationwide strategy to increase routine testing, identify new infections, and link HIV-infected individuals to care and treatment [1]. The Centers for Disease Control and Prevention (CDC) estimates that 34% of new HIV infections occurred in individuals aged 15–29 in 2009 [2]. When stratified by transmission category, greater than 60% of new cases of HIV were among men who have sex with men (MSM). While overall incidence has remained stable, HIV rates in those aged 13–29 increased by 21% from 2006 to 2009, with an even more disproportionate 48% increase among young, black MSM [3,4]. A similar epidemiology has been shown among syphilis, with 63% of new infections occurring in MSM and increasing rates in the 15–24 age group during 2004–2008. This suggests a rate of HIV and syphilis incidence that is at least 40 times greater in MSM than other risk groups and mainly in younger individuals [5].

In 2010, there were approximately 12.1 million full-time enrollees in 2,348 four-year colleges and universities throughout the United States [6]. This population is primarily comprised of adolescents and young adults, a group that offers unique challenges for HIV and syphilis prevention. Students are often characterized as being sexually active and having high-risk behaviors, such as multiple sexual partners and inconsistent condom use [7-11]. Students also tend to believe that they are at little to no risk of contracting HIV [12,13]. The age demographic and behaviors of college students place them at risk for infection with HIV and syphilis. Previous studies from colleges in the Southeastern United States demonstrated that a high percentage (>10%) of new infections were among college students [14,15]. These college students tended to be MSM and African American. Students at-risk for infection were found to have perceptions of low personal risk of HIV, believing that HIV dialogue may be detrimental to their relationships [16]. Despite these findings, there have been few public health efforts or follow-up studies to prevent further HIV transmission on college campuses.

In November of 2010, three new cases of acute/recent HIV infection were reported in college students in Rhode Island [17]. These cases, which were diagnosed at local college health centers, suggested there was ongoing transmission in the college community, specifically during the acute phase of HIV when antibody testing can be negative and the risk of transmission is increased [18]. At least one student specifically requested an HIV RNA test (viral load) when his antibody test was negative. Further evaluation of new HIV diagnoses in 2010 in our community revealed a high prevalence of academic students (17%) of which over 70% were MSM and most presented with acute or primary HIV infection (infection within the last six months) [17].

Acute HIV infection is the period of time from infection to the development of antibodies, which is generally two or three weeks but can be as long as several months. Approximately 50 to 80% of individuals with acute HIV infection present with non-specific flu-like symptoms. Standard HIV testing of the serum and oral saliva both involve testing for antibody formation. Thus, an individual with acute HIV infection may have a negative antibody test. Furthermore, acute HIV infection is often misdiagnosed as another viral illness [19]. Medical providers must have a high index of suspicion to diagnose acute HIV infection. The diagnosis is made by testing for HIV RNA (viral load) which must be run at specialized laboratories. Acute HIV infection may comprise a significant number of new infections which are missed by standard antibody tests [18,20].

The prevalence of HIV in the general college population was low in early epidemiological studies [21]. Given the current data suggesting increasing rates in younger populations, evaluation of current testing practices on college campuses is needed to address this shift in demographics. College health services serve as an important source for primary and preventative care for students. Effective HIV and sexually transmitted infection (STI) testing practices and education may prevent further infections both during the college years and later in life. To our knowledge, there is little current data regarding testing practices of HIV and other STIs on college campuses, experiences with acute HIV infection, and the approaches toward addressing sexual healthcare needs in MSM. These issues are critical in designing effective HIV and STI prevention programs on college campuses and addressing the growing epidemic of HIV among younger adults and MSM. This report describes a preliminary survey of college health services in New England regarding their experiences with HIV and STI testing.

## Methods

We performed a cross-sectional survey via phone interview on HIV/STI testing practices during the 2010–2011 academic year. The interview was conducted with medical directors at New England college health centers during June to September of 2011. Our sample was restricted to New England’s 4-year colleges with non-commuter campuses assuming these schools were more likely to have full-time student health centers with primary health and preventative care services. There were 130 schools that met our inclusion criteria. Characteristics of schools were compiled including size (based on full-time enrollment), private versus public, geographic location, urban versus non-urban, and religious affiliation. Quota sampling was used to identify a representative sample totaling 59 schools based on these characteristics. Recruitment e-mails and letters were sent to medical directors at selected schools. Health centers were subsequently contacted to ask if the medical director was willing to participate. Three attempts were made to contact each school by interviewers.

Categorical questions on the survey were based on the American College Health Association’s (ACHA) annual Pap Test and STI Survey which has been extensively piloted in the college setting [8]. These included yes/no questions on whether HIV, syphilis, and other STI testing was offered at the student health center, whether written consent was required, if anonymous testing was available, or if an appointment was needed. Other categorical questions included cost of testing, mode of HIV testing (i.e. rapid versus serum), how results were reported, and how student health centers offer STI testing (routine, on request, specific groups such as MSM, clinical suspicion, etc.). The remaining questions were conducted in a qualitative manner and included open-ended questions about general HIV testing, acute HIV infection, other STI testing, and outreach efforts targeting MSM and other high-risk groups. Questions about gonorrhea and chlamydia were included as a reference to compare HIV and syphilis testing practices against. The study was submitted to the Brown University Institutional Review Board, which deemed it exempt from “human subjects research” as defined in Title 45 CFR Part 46.

Aggregate data was compiled from survey participants. Common themes were highlighted if two or more schools shared similar concerns or ideas in response to open-ended questions. Characteristics of college health centers (school size, private/public status, geographic location, urban/non-urban, and religious affiliation) and categorical response data was analyzed using Fisher’s exact test to determine factors associated with HIV testing. Significance was defined as p-values less than 0.05.

## Results

A total of 31 medical directors participated in this study (response rate of 52.5%). Among these schools, 58% (18/31) had a full-time enrollment of greater than 5,000 students and 74% (23/31) were private institutions (Table 1). A total of 16% (5/31) reported a religious affiliation, all of which was Catholic. Approximately 55% (17/31) were situated on a non-urban campus. All six states of New England were represented with 10 schools from Massachusetts, seven from Rhode Island, six from Connecticut, four from New Hampshire, three from Vermont, and one from Maine.

**Table 1:** Characteristics of colleges and universities (N = 31)

	
≥ 5,000 students	58% (18/31)
< 5,000 students	42% (13/31)
	
Connecticut	19% (6/31)
Massachusetts	32% (10/31)
Maine	3% (1/31)
New Hampshire	13% (4/31)
Rhode Island	23% (7/31)
Vermont	10% (3/31)
	
Public	26% (8/31)
Private	74% (23/31)
	
Urban	45% (14/31)
Non-Urban	55% (17/31)
	
Religious Affiliation	16% (5/31)
Nonsectarian	84% (26/31)

### HIV testing

Over 90% (28/31) of schools provided some form of HIV antibody testing (Table 2). Among the 28 schools that offered HIV antibody testing, a total of 7,533 tests (Range: 1–1500) were performed during the 2010–2011 academic year. The total full-time enrollment of all participating schools that offered HIV antibody testing was 212,910 students, as reported by the National Center for Education Statistics [6]. This corresponds to a testing rate of 3.54% in the past academic year (95% CI: 3.46-3.62). More than half of health centers tested less than 2.5% of the total student body. During the 2010–2011 academic year, a total of four antibody tests were positive and subsequently confirmed for HIV.

**Table 2:** HIV testing practices at student health centers during the 2010–2011 academic year (N = 31)

	
HIV antibody testing offered	
Yes	90% (28/31)
No	10% (4/31)
Rapid HIV antibody testing offered	
Yes	38% (10/26)
No	62% (16/26)
Failure of students to receive results	
Never	53% (9/17)
Infrequently	41% (7/17)
Sometimes	6% (1/17)
Number of HIV tests	
Total	7537
Mean (n = 28)	269
Number of positive tests	
Total	4
Cost of HIV testing (no insurance)	
Mean	$26 ($12.50 - $65)
Median	$25
Written consent required	
Yes	43% (12/28)
No	57% (16/28)
Anonymous testing offered	
Yes	21% (6/28)
No	79% (22/28)
Appointment needed	
Yes	68% (19/28)
No	32% (9/28)
Post-exposure prophylaxis (PEP) available	
Yes	34% (10/29)
No	66% (19/29)
Comfortable with recognition of acute HIV infection	
Yes	71% (22/31)
No	29% (9/31)
Diagnosis of acute HIV infection (ever)	
Yes	16% (5/31)
No	84% (26/31)
Viral load testing available	
Yes	70% (21/30)
No	30% (9/30)

No schools had clear guidelines indicating which students should or should not be offered HIV antibody testing. No schools routinely tested all students or provided opt-out testing (practice that includes routine HIV testing unless individual specifically declines). However, 96% (27/28) of schools which offered HIV antibody testing did so to any student who specifically asked to be tested. All schools offering HIV antibody testing provided testing if a healthcare provider suspected HIV based on clinical presentation or in cases of sexual assault. Most schools offered HIV antibody testing during routine women’s health visits, specifically during gynecologic visits or visits to discuss contraception. Three-quarters of schools routinely tested MSM for HIV although many medical directors remarked that the decision about testing is primarily based on self-reported risk behaviors of the individual students.

Of schools that performed HIV antibody testing, 68% (19/28) required an appointment for testing, although nearly all of those requiring appointments stated same-day appointments were available if requested. Urban schools were more likely to require an appointment for HIV testing compared to non-urban schools (79%, 11/14 urban schools required an appointment versus 47%, 8/17 non-urban, p-value = 0.15). Additionally, 90% (25/28) of health centers only provided testing services at the on-campus health center or laboratory. The remaining schools provided either yearly or twice yearly testing at off-campus sites, mostly sponsored by the local campus health center. Written consent for HIV testing was required at one-third of colleges, many of which were in Massachusetts where it is required by state law. Other schools in Connecticut and New Hampshire required written consent as a school policy. Anonymous testing was available at 21% (6/28) of sites. With respect to the type of testing that was offered, 32% (10/31) of respondents stated that rapid antibody testing was available. Schools larger than 5,000 students tended to offer rapid testing more often than did smaller schools (44%, 8/18 schools larger than 5,000 students offered rapid testing versus 15%, 2/13 smaller schools, p-value = 0.18), while schools having a religious affiliation tended not to have rapid testing available compared to nonsectarian schools (0%, 0/5 religious schools offered rapid testing versus 38%, 10/26 nonsectarian schools, p-value = 0.24). Of those schools that did not provide rapid antibody testing, nearly all (91%, 16/18) required students to return to the health center for their results with only one school allowing students to receive results over the phone. Although nine schools did report at least one case of a student not returning for HIV test results during the 2010–2011 academic year, this was a rare occurrence. With respect to cost of testing, 14% (4/28) of schools provided free HIV antibody testing to all students, regardless of insurance status. All of the remaining schools offering testing stated that student insurance covered the cost of the associated appointment and testing but that there was co-pay ranging from $5 to $80. The mean out of pocket expense when not using insurance was $28.78 (median $25) for an HIV antibody test and $90.57 (median $48) for an STI panel covering chlamydia, gonorrhea, and syphilis.

The primary barriers to HIV testing identified by medical directors were financial including cost to students and lack of resources and support services to promote and offer testing. Other barriers included difficulty engaging students and having them present to the health center for testing, “complacent” attitudes about HIV testing, low risk perceptions for HIV infection, stigma, and structural barriers (need for appointments, lack of rapid testing).

### Acute HIV infection

Seventy-one percent of medical directors stated their providers are comfortable with the recognition and diagnosis of acute HIV infection, although many directors described a need to provide further training regarding acute HIV infection on campus. Schools with a religious affiliation reported less comfort with the recognition and diagnosis of acute HIV infection when compared with nonsectarian schools (20%, 1/5 affiliated schools versus 81%, 21/26 nonsectarian, p-value < 0.05). A modest 16% (5/31) of schools reported ever diagnosing a case of acute HIV infection, with five schools over four different states in New England.. In total, 58% (18/31) of survey respondents stated their providers had received some training in the recognition and diagnosis of acute HIV infection. This training was primarily in the form of on-campus presentations from faculty at neighboring institutions and was reported as having occurred during training or residency or during in-service presentations more than five years ago. There was a particular need to train providers in differentiating which students needed testing immediately and those that could be safely observed.

Most respondents (67%, 21/31) stated their health center could perform HIV RNA testing for students for whom acute HIV infection was clinically suspected. Schools in urban settings were significantly more likely to offer HIV RNA (viral load) testing than those in non-urban settings (93%, 13/14 urban setting schools offered testing versus 47%, 8/17 non-urban, p-value = 0.02). Urban schools also tended to offer post-exposure prophylaxis (PEP) as compared with non-urban schools (50%, 7/14 urban schools offered PEP versus 18%, 3/17 non-urban, p-value = 0.13).

### Testing for other sexually transmitted infections

Ninety-four percent (29/31) of student health centers performed testing for other STIs including gonorrhea, chlamydia and syphilis (Table 3). Ninety-seven percent (28/29) of schools that provided STI testing would perform testing on students who asked for it, who have been sexual assaulted, or when a specific STI was clinically suspected. In contrast to HIV antibody testing, at least 25% (8/29) of schools had clear clinical guidelines about when to perform STI testing. A common example guideline included ‘all women who receive Pap smears will have chlamydia and/or gonorrhea testing’. There was less of a consensus about routine testing in men and MSM for STIs. Approximately 62% (18/29) of schools routinely tested MSM for gonorrhea, chlamydia and syphilis, while the other schools tested based on clinical presentation or patient-generated requests.

**Table 3:** Sexually transmitted infection (STI) testing practices at student health centers (N = 31)

	
STI testing offered*	
Yes	94% (29/31)
No	6% (2/31)
Cost of STI testing	
Mean	$94 ($0-225)
Median	$56.50
Appointment needed	
Yes	69% (20/29)
No	31% (9/29)
Conduct outreach to high-risk groups	
Yes	71% (22/31)
No	29% (9/31)
Targeted educational resources for LGBT	
Yes	77% (24/31)
No	23% (7/31)
Training on LGBTQ healthcare	
Yes	74% (23/31)
No	26% (8/31)

*Includes gonorrhea, chlamydia, and syphilis testing.

LGBT = Lesbian, gay, bisexual, transgender.

Ninety-four percent (27/29) of student health centers performed STI testing on-campus and only one school provided urinary gonorrhea and chlamydia testing at a once-yearly, off-campus site. Similar to HIV, 69% (20/29) of schools required an appointment for STI testing. With respect to cost of testing, two schools provided free testing, while 94% (22/29) of schools provided appointments and testing that were covered by student insurance, with a co-pay ranging from $0 to $35.

### Targeted outreach

Seventy-seven percent of college health centers had ever conducted training for providers about specific lesbian, gay, bisexual and transgender (LGBT) sexual healthcare needs. Topics included sensitivity training about transgender healthcare concerns, appropriateness of language used by providers in addressing LGBT students, and techniques for gathering a detailed sexual history from LGBT students. A number of directors commented that their training modules may be insufficient, noting there was a particular need to help train providers with sexual history gathering for all students and more specifically for LGBT students.

Seventy-seven percent of college health centers performed some form of targeted outreach to at-risk groups, specifically by reaching out to LGBT groups on their respective campuses. Fewer schools with a religious affiliation tended to conduct outreach compared to nonsectarian schools (40%, 2/5 affiliated schools conducted outreach to high-risk sexual groups versus 77%, 20/26 nonsectarian, p-value = 0.13). The majority of respondents reported that they either handed out brochures and pamphlets to these groups with targeted information, or delivered on-campus presentations about the need to get tested for HIV and STI testing and methods for practicing safer sex. Many health directors described a need for further outreach efforts as well, with at least two directors noting that while they had tried performing targeted outreach, their own efforts were “few and far between”. Some individuals also commented that the targeted pamphlets they provide were outdated and weren’t “appealing enough to be read by students”.

While some providers recognized many LGBT students would benefit from testing more than once a year, they were unclear about which students required more frequent testing. Furthermore, a number of medical directors commented that it was difficult to identify the LGBT students on campus. This was particularly common at schools with a religious affiliation, which noted that perceptions that the health centers were unwelcoming made it difficult to provide targeted outreach efforts. In particular, health centers at religiously affiliated schools reported low HIV risk perception on campus, the inability to distribute condoms, and the inability to conduct sexual health outreach activities as a direct result of the religious affiliation. When asked about outreach to at-risk groups, one medical director responded, “we don’t have a high MSM population, this being a Catholic institution,” instead the director said the center “focused on inclusivity.”

## Discussion

### HIV testing

Our results reveal that there is widespread under-testing of HIV on college campuses throughout New England. The cumulative testing rates were less than 5% which excludes the 18,303 students at participant schools where HIV testing is not offered. Despite these low testing rates, four cases of HIV were diagnosed at New England college health centers during the 2010–2011 academic year. This is a conservative estimate based on recall bias of the medical directors we interviewed. According to the revised CDC recommendations, HIV screening is recommended for all patients in an opt-out format, particularly in those individuals who have never been tested [22]. The CDC recommends repeat annual testing for those individuals who are at high-risk, including MSM and heterosexual persons who have had more than one sexual partner since their most recent HIV test. These guidelines suggest that college students should be tested at least once for HIV and in many cases annually. This is in contrast to the small minority of college students who report being tested for HIV at least once in their lives. Rates of HIV are increasing among younger MSM and these recommendations should be strongly encouraged on college campuses to ensure MSM participate in HIV testing campaigns.

College health services represent a unique venue to promote HIV testing among students, especially as students make visits to a college health center more than once annually on average. These centers also represent an opportunity to educate students about safe sexual and testing behaviors. Many medical directors commented that students and providers alike have become “complacent” about HIV on college campuses. Directors feel that students’ reasons for being HIV tested have morphed from a time in the late 1980’s when people feared being infected with HIV to a “routine activity” for a handful of diligent students in the early 2010’s. Given the infrequency of reported cases on college campuses, directors describe losing momentum in addressing HIV preventative care on campus.

### Acute HIV infection

Acute HIV infection can be difficult to diagnose and requires a high index of suspicion. Many providers are not comfortable recognizing the clinical manifestations of acute HIV infection. This is likely an underestimate given reporting bias. The discomfort with recognizing, and the lack of training and testing for, acute HIV infection represents a number of missed opportunities for HIV diagnosis and prevention. Rates of transmission are higher in patients during the acute phase of HIV when viral loads are highest and many new cases can be attributed to persons during this period [18]. Additionally, individuals who find out they are HIV-infected, subsequently reduce high-risk sexual behaviors following a diagnosis. Medical providers on college campuses should take a sexual history from students presenting with flu-like symptoms. Acute HIV infection should be suspected in the setting of recent (within the last 2–3 weeks) episodes of unprotected sex, especially in MSM and students with multiple or anonymous partners. Students should be counseled that a negative HIV antibody test at this time does not necessarily rule out HIV infection. An HIV RNA (viral load) may be sent to confirm a diagnosis of acute HIV infection. Alternatively, HIV antibody testing may be performed at one month, three month, and six month intervals.

Given that up to 90% of patients may experience some form of acute illness before reaching a latency phase of HIV [18], it is important to increase provider knowledge of acute HIV through training in a variety of healthcare settings, including college health centers. Many directors specifically requested further training regarding acute HIV symptom recognition and testing protocols to help determine which students would benefit from testing.

### Barriers to HIV and STI testing

Many college health directors have begun addressing various barriers to HIV, gonorrhea, chlamydia and syphilis testing. These barriers include cost, availability of testing, and the need for appointments versus walk-in testing. The latter has been addressed at some schools by having on-campus testing at sites other than the health centers such as health fairs in the local campus center. This has led to new venues for testing while still providing valuable time for patient education. For HIV testing, some schools have implemented rapid testing which directors uniformly agree is significantly easier and has significantly improved testing practices and education within the clinic, particularly since students no longer need to return for results. Anonymous testing was uncommon at most health centers, though many have attempted to develop confidential testing methods that, for example, do not provide information about the specific laboratory tests performed on laboratory billing invoices. Based on our qualitative results, the clearest variable to cause sudden and dramatic improvements in HIV testing rates was the availability of free HIV testing, regardless of insurance status. Schools that provided free testing had higher total numbers of tests and higher rates of testing as a percentage of the study body population. Due to resource limitations, one school has begun providing free testing only at venues aimed at LGBT groups, due to the increased rate of HIV in this demographic.

### Targeted outreach

Outreach efforts at student health centers targeting high-risk sexual groups are encouraging. Many directors highlighted this important need, but also mentioned that their efforts may not be sufficient. Many centers described a need for more LGBT-specific training modules for providers particularly in gathering detailed sexual histories from this subset of patients. Given the recent CDC data showing the HIV epidemic moving into younger MSM populations, providers at student health centers may readily serve as important and existing infrastructure to perform testing. Investment in this training may have widespread benefits in addressing HIV prevention among younger populations in the United States.

Medical directors uniformly agreed that it was important to find venues to engage with LGBT populations, particularly MSM, to discuss their distinct sexual healthcare needs. These comments were often coupled with a desire for clearer guidelines regarding HIV and STI prevention in LGBT populations.

There were a number of limitations to this study. First, the sample size was small (31) and the response rate of the survey was low (52.5%). Although not all schools in the region were captured, the study is the first to start to systematically evaluate HIV and STI testing practices at college health centers in our region. Second, the study was restricted to New England. While demographics of students may change from region to region, college students in New England often represent students from all over the United States. Third, the results were provided by medical directors at their discretion and may be subject to recall bias. It is possible that larger schools with better data systems, staffing and resources that were comfortable with their HIV and STI testing practices were more likely to respond quickly to participation inquiries. The responses of the medical directors may not represent all individuals at the entire health center and should be taken into consideration.

College health services are an integral part of student health on many college campuses. Recent surveillance data suggests an increase in HIV infection in college-aged populations, specifically MSM. Effective HIV prevention programs on college campuses should encourage HIV testing for all college students, with targeted efforts for MSM. Barriers to HIV and STI testing such as cost and availability should be addressed and minimized. Medical providers who provide care to college students should be able to recognize the signs and symptoms of acute HIV infection such as individuals presenting with flu-like symptoms after a high-risk sexual exposure. Student health centers have the potential to provide effective and targeted HIV testing programs on college campuses.

## Competing interests

The authors declare that they have no competing interests.

## Authors’ contributions

NP, AT, TPF, and AR were involved in designing and conducting the study. PAC, JCB, NP, AT, TPF, JJV, and AR were all involved in the analysis of results, writing and editing of the manuscript. All authors read and approved the final manuscript.
